# Moral Obligation, Public Leadership, and Collective Action for Epidemic Prevention and Control: Evidence from the Corona Virus Disease 2019 (COVID-19) Emergency

**DOI:** 10.3390/ijerph17082731

**Published:** 2020-04-15

**Authors:** Liu Yang, Yang Ren

**Affiliations:** School of Public Affairs and Administration, University of Electronic Science and Technology of China, Chengdu 611731, China; yangliu@uestc.edu.cn

**Keywords:** moral obligation, public leadership, collective action, COVID-19

## Abstract

To investigate the effect of villagers’ moral obligation and village cadres’ public leadership on villagers’ collective action for epidemic prevention and control, against the background of the corona virus disease 2019 (COVID-19) emergency in China, we constructed models based on the institutional analysis and development (IAD) framework and employed principal component analysis (PCA) and ordered probit regression, drawing on survey data from 533 villagers in Henan province adjacent to the COVID-19 origin province, Hubei, China. The results indicate that: (1) generally, both moral obligation and public leadership as well as their constituent indicators contributed positively to collective action for COVID-19 prevention and control; (2) moreover, moral obligation and public leadership can strengthen each other’s positive role in collective action for COVID-19 prevention and control. Based on the above findings, this paper suggests that villagers’ moral obligation can be perfected through internalizing epidemic prevention and control norms into the villagers’ moral norms by the way of villagers mastering the rural public health governance scheme. In addition, public leadership can be improved through professional training of village cadres and by motivating village elites to run for village cadres. With improved villagers’ moral obligation and village cadres’ public leadership, collective action for epidemic prevention and control could be more likely to be realized.

## 1. Introduction

On 12 December, 2019, the 2019 novel corona virus (2019-nCoV) emerged in Wuhan, Hubei province, China. It caused a worldwide epidemic, corona virus disease 2019 (COVID-19), in humans mainly through respiratory transmission. Within 77 days, 2019-nCoV had spread to all China’s 34 provinces and municipalities, infected 78,064 Chinese residents and killed 2715 of them (by 26 February, 2020) [1], although Chinese central and local governments had taken strict prevention and control measures across the whole country. Exhorting residents to reduce the frequency with which they go outside and to wear masks during outings were the vital measures that the governments adopted. Essentially, residents’ collective response measures for epidemic prevention and control, like going outside less often and wearing masks during outings, are a kind of residents’ collective action, which depends on their behavior preferences. Residents’ collective action for epidemic prevention and control is a vital and effective measure to conquer epidemic emergencies. This is because residents’ collective action, such as reducing the frequency of going outside and wearing masks collectively, can cut off the transmission route of the epidemic and avoid cross infection efficiently. Thus, identifying the primary factors promoting collective action is necessary.

As a result of individual rational choice, individuals’ motivation to participate in collective action is affected by many factors. Numerous existing studies focus on the impact of social capital on residents’ participation in collective action [2,3,4,5,6]. They believe that social capital, such as social trust, relationship networks, and social norms, can realize the combination of residents’ micro-individual and macro-collective actions that can effectively avoid free-riding problems and promote the success of collective action [7]. Social capital mainly functions through two paths. One is to promote the individual sharing of information and achieve resource integration through individuals’ embedded social networks and social trust, thereby reducing the uncertainty of choice [8,9]. The second is to restrict and guide individual behaviors through social norms, thereby suppressing the occurrence of opportunism [10]. Social norms and moral obligations have similar influence mechanisms on collective action, but there are still large differences. Social norms mainly influence individuals’ behaviors through external pressure and external behavioral supervision [5]. Moral obligation is the internalization of good behavior norms by individuals, which mainly affects individual behavior through internal constraints [11]. Studies show that people’s internal ideology can significantly affect their cost–benefit comparisons and collective action choices [12], but few examine collective action in epidemic prevention and control from the perspective of moral obligations.

Some scholars investigated the role of the government in residents’ collective action in public governance [13]. They believe that governments’ policy and financial support are beneficial for the construction of various cooperative organizations of residents [14]. These organizations can increase the possibility of residents participating in collective action through frequent information communication and cooperation. Moreover, the government’s guidance and support reduce the input costs of residents’ participation in public governance; thus, they can motivate residents to participate in collective action [15]. However, the effect of government support does not depend entirely on the support policy itself, but more on the implementation of policies by local leaders. This is because good leaders can use their personal influence and position to transform the policy designed at the top into specific governance measures at the grassroots level, thereby improving the ability of public governance at the grassroots level [16]. Unfortunately, at present, although some scholars are concerned about the positive role of leadership in collective action, public leadership is rarely researched in relation to collective action at the grassroots level. In addition, the relationship between public leadership and collective action for epidemic prevention and control is not part of scholars’ visions.

Residents’ moral obligation and local leaders’ public leadership are theoretically two key factors that affect the generation of residents’ collective action. On the one hand, individuals’ moral obligation could promote their commitment to action according to their conscience, no matter how much it may cost or whether it is likely to succeed [17]. Thus, residents with moral obligation normally tend to contribute to collective action by following exhortations for collective action and sacrificing their own utilities [17,18]. On the other hand, local leaders’ public leadership can enable the measures related to collective action to be improved and implemented adequately, thus constraining residents’ harmful behaviors and facilitating their beneficial behaviors [19]. Besides, moral obligations and public leadership can play a synergistic role in affecting residents’ collective action. However, the aforementioned theoretical logic of moral obligation and public leadership affecting residents’ collective action is not systematically analyzed empirically in prior research. Therefore, this study strives to fill this academic gap by clarifying the effects of moral obligation and public leadership on residents’ collective action to address epidemic prevention and control. Moral obligations can increase residents’ awareness of participating in collective action for epidemic prevention and control, while public leadership mainly promotes the success of collective action by coordinating the relationships between stakeholders to ensure orderly participation behavior. Therefore, in collective action research, the introduction of moral obligation and public leadership has important theoretical and practical significance. The results of this study could thus offer some policy implications for promoting the necessary collective action for epidemic prevention and control.

According to the above analysis, theoretically, villagers’ moral obligation can directly affect their behavior preferences in collective action. Besides, in villages, the leaders of the village committee are the main implementers of the government’s policies and requests. Therefore, villagers’ reaction to collective action for epidemic prevention and control exhorted by the government also depends to some extent on the leaders’ public leadership. In rural China, since the village committee is a self-governing organization and the public health system is relatively weak, epidemic prevention and control rely heavily on villagers’ collective action. Therefore, China’s village collective action to conquer the COVID-19 emergency provides the opportunity to conduct scientific research to analyze the effect of moral obligation and public leadership on collective action for epidemic prevention and control. Therefore, based on the institutional analysis and development (IAD) framework, this study mainly employs ordered probit regression using survey data from 533 villagers in the rural Henan province, adjacent to the COVID-19 origin province, Hubei, China, to investigate empirically the effects of villagers’ moral obligation and village cadres’ public leadership on villagers’ collective action for COVID-19 prevention and control.

## 2. Theoretical Framework

### 2.1. Effects of Moral Obligation and Public Leadership

#### 2.1.1. Effect of Moral Obligation

Villagers’ collective action for epidemic prevention and control can be affected not only by external governmental prevention and control measures but also by villagers’ internal factors. Moral obligation is a crucial factor among these villagers’ internal factors. Essentially, moral obligation is part of the people’s internalized value evaluation system [20] and is normally defined as “a personal decision to participate in a specific collective action based on the belief that this is what should be done” [21]. Since moral obligation could offer an internalized incentive for villagers to participate in collective action, it plays a key role in facilitating the spontaneous maintenance of social order [22,23]. Based on the existing research, we propose that villagers’ moral obligation affects collective action for epidemic prevention and control through two principle theoretical paths: on the one hand, villagers with moral obligation normally tend to take various epidemic prevention and control measures [24], such as reducing the frequency of going outside, wearing masks, and refusing to attend parties, to generate collective action for epidemic prevention and control; on the other hand, since Chinese villages have an acquaintance society, villagers strongly rely on their social network [25,26]. In collective action for epidemic prevention and control, villagers who do not conduct the required measures could increase their risk of infection and spreading the epidemic. These destructive behaviors would be condemned by other villagers, which would lead to the corruption of villagers’ reputation and an acquired-trust crisis [27]. Even worse, these immoral “rule breakers” would be excluded from the village network. The abovementioned predictable negative consequences of villagers’ immorality in epidemic prevention and control could increase their psychological stress and then suppress their immoral behaviors. Thus, we propose that villagers’ strong moral obligation could motivate them to constrain their immoral behaviors better and contribute more to collective action for epidemic prevention and control.

#### 2.1.2. Effect of Public Leadership

Whether epidemics can be prevented and controlled effectively in rural China depends not only on epidemic prevention and control schemes, but also on the effective implementation of these schemes. The main result of effective implementation is the successful generation of villagers’ collective action for epidemic prevention and control. However, the achievement of collective action is inseparable from the public leadership of village cadres [28,29,30]. This is because public leadership could affect villagers’ trust in public departments and organizations and thus their reaction to epidemic prevention and control scheme [31]. Public leadership refers to the leadership for the common good with the purpose of creating public value [32]. Qualified village cadres can convert their personal leadership into public leadership. Accordingly, public leadership can enable village cadres to coordinate villages’ political, economic, and social resources adequately to manage villages under the existing institution system. This is helpful in realizing villages’ collective action for epidemic prevention and control. The reason consists of two aspects. On the one hand, qualified village cadres can work as an efficient hub linking the government and villagers by enabling villagers to understand fully the government’s various epidemic prevention and control measures and relay the villagers’ reasonable demands to the government in a timely manner [33]. On the other hand, qualified village cadres can use their leadership to balance conflicts among villagers [34], which is beneficial for villagers’ adoption of epidemic prevention and control measures, and thus achieve effective collective action for epidemic prevention and control [35].

#### 2.1.3. Interaction Effects of Moral Obligation and Public Leadership

In collective action for epidemic prevention and control, moral obligations and public leadership can play a synergistic role. Moral obligation inwardly restricts villagers’ behavior of [36], and public leadership restricts their behavior by changing the external institutional environment in which they live [37]. Generally speaking, moral obligation and public leadership have different governance functions in villagers’ collective action, supporting and complementing each other. Specifically, the supply of public leadership in rural China is often inadequate [19]. In this case, moral obligations can generate a complementary effect. Villagers with strong moral obligations normally tend to respond zealously to village cadres’ calls for epidemic prevention and control [24], which can effectively reduce village cadres’ difficulties in achieving epidemic prevention and control and promote public leadership to play a more effective and significant role. In addition, villagers’ moral behavior in epidemic prevention and control can stimulate village cadres’ service consciousness. This service consciousness can theoretically motivate village cadres to supply strong public leadership. Accordingly, strong public leadership can form powerful constraints on villagers’ behaviors [38], prompting more villagers with weak moral obligations to take prevention and control measures, thereby effectively achieving collective action for epidemic prevention and control.

### 2.2. The IAD Framework

Previous studies show that multiple factors are positively correlated with collective action. The IAD framework can integrate multiple influencing factors into the same framework for collective action analysis [15]. It aims to explain how external variables affect the self-governance of common-pool resources by influencing actors’ interaction [39]. Since COVID-19 prevention and control are governance of public affairs, the main purpose of which is to achieve collective action, this paper employs the IAD framework to analyze the influential factors on collective action for COVID-19 prevention and control.

**Figure 1:**
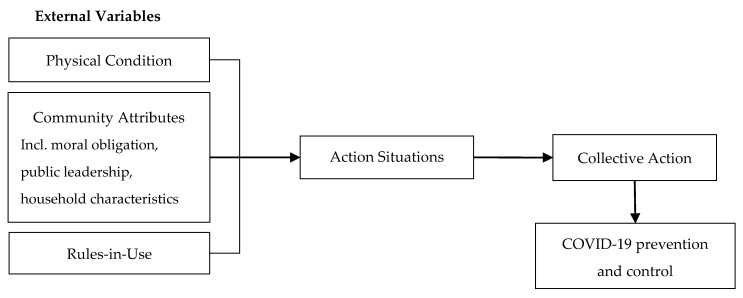
Theoretical framework to analyze the influential factors of collective action for epidemic prevention and control. Source: Adapted from Ostrom [39].

The IAD framework included external variables, action situations, interactions, and outcomes [40]. External variables can affect action situations to generate interactions and outcomes. In this paper, the interactions and outcomes are collective action along with COVID-19 prevention and control, respectively. Action situations are the core of the IAD framework. In action situations, the actors can interact, acquire resources, supervise, negotiate, solve problems, and compete or cooperate [41]. Moreover, the strategic choice of actors in action situations can be affected by external variables. Many studies focus on three categories of external variables: physical condition, community attributes, and rules-in-use [12,42]. Physical condition creates opportunities for, or can place constraints on the situation that actors face [43]. Community attributes include internal homogeneity or heterogeneity and the knowledge and capital of actors [44]. Since people’s behavior is affected by the characteristic of their families, household characteristics are considered to enrich community attributes besides moral obligation and public leadership. Rules-in-use refer to enforced prescriptions about the actions that are required, prohibited, or permitted [45]. Here, this paper focuses on external variables and how they affect collective action for COVID-19 prevention and control. Figure 1. shows the theoretical framework of the current study.

## 3. Data and Empirical Approach

### 3.1. The Study Area and Data Collection

The data used in this research were collected through an internet questionnaire survey conducted in villages located in Henan province, China. The reason for the choice of rural Henan as the research site is fourfold. Firstly, Henan province and Hubei province are adjacent. At the point of investigation, Henan province had a relatively large number of COVID-19 cases, following Hubei province. Secondly, Henan province has the largest population with the highest density of all the Chinese provinces, which could enable the epidemic to spread extremely quickly. In this situation, it is vital to have well-organized collective action, especially in villages with a relatively weak health system. Thus, research on the factors influencing the generation of collective action in this area is meaningful. Thirdly, the phenomenon of house gathering is significant in villages in Henan province because of the geographic attribution. This further increases the need for collective action for COVID-19 prevention and control. Fourth, villages in Henan province took the lead in adopting some efficient measures to stimulate the generation of collective action for COVID-19 prevention and control in China. These measures include providing villagers with knowledge related to COVID-19; publicizing the necessity not to go out; wearing masks and other protection measures; taking reasonable traffic control to constrain villagers’ sphere of activities and unnecessary transportation; implementing a punishment mechanism to suppress villagers’ behaviors that are harmful to COVID-19 prevention and control; and finding and reporting suspected infections to local government in a timely manner.

We conducted the survey for this research from February 7 to 18 February, 2020. The questionnaire was distributed through WeChat, which is mobile social software used by more than 70% of Chinese people. There is a region-filtering options in the questionnaire, which should ensure that the completed questionnaires that we received were all from rural areas in Henan province. After eliminating the questionnaires that did not meet the requirements of this research because of unreasonable or incomplete responses, 533 valid samples from Henan villagers were finally obtained.

### 3.2. Empirical Approach

This study employed ordered probit regression and principal component analysis (PCA) to analyze the data. The dependent variables, the frequencies of villagers going out and wearing masks, were measured on a five-point Likert scale. They are limited and have a natural ordering. Therefore, ordered probit regression was suitable because it can be used when the dependent variables are multiple and ranked discretely [46]. Models can be formulated as a threshold model as follows:

(1) $$y_{i}^{*}=x_{i}\;\beta^{\prime }+\varepsilon_{i}\;;\;i=1,\;2,\cdots ,\;N$$

where $y_{i}^{*}$ is a latent dependent variable; $x_{i}$ is the vector of the influential factors; $\beta^{\prime }$ is the vector of regression coefficient that we need to estimate; and $\varepsilon_{i}$ is a vector of unknown parameters with N (0, 1) [47]. Since $y_{i}^{*}$ is unobserved, we can observe only the response categories $y_{i}$. In this paper, Equation (1) can be specified as follows:

(2) $$\begin{array}{l} CollectiveAction_{i}\\ \;=\beta_{0}+\beta_{1}MoralObligation_{i}+\beta_{2}PublicLeadership_{i}\\ \;+\beta_{3}PhysicalCondition_{i}+\beta_{4}HouseholdCharacteristics_{i\;}\\ \;+\beta_{5}RulesinUse_{i}+\varepsilon_{i} \end{array}$$

Based on Equation (2), this study introduced the interaction term to examine the interaction between moral obligation and public leadership in collective action for COVID-19 prevention and control. The estimated model is shown below:

(3) $$\begin{array}{l} CollectiveAction_{i}\\ \;=\beta_{0}\;+\beta_{1}MoralObligation_{i}\\ \;+\beta_{2}PublicLeadership_{i}\\ \;+\beta_{6}MoralObligation_{i}\\ \;\times PublicLeadership_{i}\\ \;+\beta_{3}PhysicalCondition_{i}\\ \;+\beta_{4}HouseholdCharacteristics_{i}\\ \;+\beta_{5}RulesinUse_{i}+\varepsilon_{i} \end{array}$$

In addition, since moral obligation and public leadership incorporate multidimensional information, PCA was employed to simplify the information and transfer the categorical variables into numeric ones [34]. This paper used STATA 15.0 (StataCorp LP., College Station, TX, USA) for ordered probit regression and IBM SPSS 25.0 (Chicago, IL, USA) for PCA.

## 4. Variable Measurements

### 4.1. Dependent Variables

Collective action for COVID-19 prevention and control was measured through the villagers’ frequencies of going out and wearing masks when they go out after 23 January, when Wuhan, the capital of Hubei province, started to conduct strict traffic control for the COVID-19 epidemic. This is because, from this point, the media and the public started to pay attention and take measures to prevent and control COVID-19. As shown in Table 1, the respondents, on average, went out once every four to seven days during the observed period. This frequency is far lower than their ordinary frequency of going out during the Chinese Spring Festival, indicating that many respondents had taken some COVID-19 prevention measures. Moreover, the mean of respondents’ frequency of wearing masks when they went out during the observed period was 2.852. Many respondents did not wear masks for two reasons. One was that masks were in short supply during the COVID-19 epidemic. Many respondents could not buy enough masks in time. The other reason was the respondents’ weak sense of prevention. They believed that there were no people who were at high risk of COVID-19 infection around them, such as people returning home from Hubei province, thus reducing the awareness of prevention measures.

### 4.2. Focused Independent Variables

This paper mainly focused on the influence of villagers’ moral obligation and village cadres’ public leadership on villagers’ collective action for COVID-19 prevention and control. The measurement scale of moral obligation was based on the study by Vilas and Sabucedo [21]. The constituent indicators used were the sense of obligation, personal satisfaction, autonomy, and objectivity. These indicators were measured on a five-point Likert scale (from 1—totally disagree to 5—totally agree). As shown in Table 1, the mean of these indicators ranged from 3.927–4.242, indicating that villagers had quite a high level of moral obligation.

Public leadership was assessed using four items adapted from the study by Han [35]. The constituent indicators were influence force, decision-making ability, executive ability, and creativity. We measured these indicators on a five-point Likert scale (from 1—totally disagree to 5—totally agree). As shown in Table 1, the mean of these indicators was from 3.233–3.700, indicating that villagers had a high evaluation of the cadres’ public leadership of their village.

### 4.3. Control Variables

The control variables were chosen based on the IAD framework. As previously mentioned, they were composed of physical condition, household characteristics and rules-in-use. Physical condition was represented by the distance from villagers living in villages to the center of their counties. It was measured on five levels. As shown in Table 1, the average distance was between 20 km and 40 km. The variables of household characteristics included respondents’ age, their education and whether a child or elderly person was living at home. The reason for choosing the item of a child or elderly person living at home was that the immunity of children and the elderly is relatively weak. Respondents might tend to adhere to COVID-19 prevention measures more strictly to protect the health of these family members. The mean of respondents’ age was 42.993. Their average education level was between “middle school” and “high school.” Of the respondents’ households, 32.3% had a child or elderly family member. In addition, the variables of rules-in-use included publicity measures, supervision mechanisms, and punishment mechanisms. Their means were all above 3.5, indicating that the rules-in-use were at a high level.

## 5. Results

### 5.1. Reliability and Validity

The reliability and validity of the variables of moral obligation and public leadership are directly related to the scientificity and effectiveness of the evaluation results of our model. Reliability is commonly tested using Cronbach’s Alpha. As shown in Table 2, the Cronbach’s Alpha values of moral obligation and public leadership were 0.817 and 0.803, respectively. Both exceeded the recommended value of 0.7. Validity analysis includes convergent validity and discriminant validity. Convergent validity is assessed through the composite reliability (CR) and the average variance extracted (AVE). The CR (acceptable level is 0.6) and AVE (acceptable level is 0.5) of moral obligation and public leadership were higher than the acceptable levels. Discriminant validity analysis is used to show whether latent variables are different from each other. The correlation coefficient between moral obligation and public leadership was 0.378, which is less than the square root of the AVE for each latent variable. In addition, the results showed that the Kaiser-Meyer-Olkin (KMO) test exceeded the recommended value of 0.7. Bartlett’s test was significant (*p* < 0.01). The indicators were above the threshold level of 0.7 for each factor loading [48]. The cumulative percentage variance (CPV) of moral obligation and public leadership was 64.962% and 63.524%, respectively. Generally speaking, the indicators of moral obligation and public leadership were highly reliable and had good validity.

### 5.2. Multicollinearity Test

This study tested multicollinearity among the explanatory variables using variance inflation factor (VIF). As shown in Table 3, the VIF value was less than 3, which means that there was no multicollinearity [49]. The ordered probit regression in this paper was correctly specified.

### 5.3. Ordered Probit Regression Analysis

The results of the ordered probit regression are presented in Table 4 (the dependent variable is going out) and Table 5 (the dependent variable is wearing masks). The independent variables in models 1 and 5 are general moral obligation and general public leadership. Control variables were added in models 2 and 6 based on model 1 and 5, respectively. In models 3 and 7, moral obligation and public leadership were represented by their constituent indicators. Models 4 and 8 added the interactive item based on models 2 and 6, respectively. All the models (LR χ^2^) were highly significant (0.0000), with the pseudo R^2^ ranging from 0.1139—0.1568. This indicates the robustness of the variables included in the model. 

Moral obligation and its constituent indicators contributed positively to collective action for COVID-19 prevention and control. The effects of general moral obligation and autonomy were significant. Besides, objectivity only had a significant impact on villagers’ going out frequency. In addition, public leadership and its constituent indicators had a positive effect on collective action for COVID-19 prevention and control. General public leadership and executive ability had a significant effects on villagers’ going out frequency. General public leadership, influence, and decision-making ability had significant impacts on villagers’ wearing of masks. Moreover, the interactive item of general moral obligation and public leadership had significantly positive effects on collective action for COVID-19 prevention and control. This indicated that moral obligation and public leadership can strengthen each other’s positive role in collective action for COVID-19 prevention and control. Among the control variables, distance to the county significantly and positively contributed to collective action for COVID-19 prevention and control. Besides, publicity had a significantly positive effect on villagers’ going out frequency during the COVID-19 epidemic.

## 6. Discussion

The main goal of this paper was to analyze the effects of villagers’ moral obligation and village cadres’ public leadership on villagers’ collective action for epidemic prevention and control. To achieve this goal, measurement scales of moral obligation and public leadership were developed, and the interactive items of moral obligation and public leadership were introduced into the model. Our results confirm that both focused independent variables played a positive role in collective action for COVID-19 prevention and control. This is consistent with the studies by Sabucedo et al. [11] and Shu et al. [19], although the scenarios of our study were different from theirs. Furthermore, moral obligation and public leadership can promote each other’s positive roles in collective action for COVID-19 prevention and control.

The possible explanations for the positive role that moral obligation can play in COVID-19 prevention and control are as follows. In rural Henan, the villagers live in close quarters and know each other very well [50]. Under the internal moral constraint of taking responsibility for themselves and their neighbors, villagers tended to take protective measures during the COVID-19 epidemic. Besides, villages in Henan province are a society of acquaintances [25]. Information can spread rapidly through frequent contact among villagers. In this situation, immoral behavior is quickly known to other villagers. Immoralists may be condemned by public opinion and even institutional punishment [51]. These constraints could reduce the probability of villagers engaging in immoral behavior and prompt them to participate in collective action for COVID-19 prevention and control.

The positive role of public leadership in collective action is closely related to a series of prevention and control schemes implemented by village cadres during the COVID-19 epidemic. Firstly, village cadres strictly screened people returning from outside the village, especially from Hubei province, in January 2020. This was not only because COVID-19 started to spread sharply across the whole of China in January 2020, but also because the returnees had the highest risk of carrying 2019-nCoV and bringing it back to rural Henan province, where there were originally no 2019-nCoV. Secondly, to prevent the returnees from carrying 2019-nCoV and causing others to be infected, the village cadres persuaded the returnees to stay at home and try not to approach the surrounding villagers. Moreover, village doctors were arranged to take the temperature of the returnees every day and monitor their physical conditions. Once the returnees showed suspected COVID-19 symptoms, they would be sent immediately to the designated hospital for isolation treatment. Thirdly, the spreading of this epidemic occurred around the time of the Chinese Spring Festival, when Chinese people traditionally visit relatives and friends. To reduce the risk of cross infection, village cadres urged villagers to stay at home and not visit relatives and friends. In addition, supervisory personnel were set up at the main exit and entrance of the village. For villagers who wanted to leave the village, the supervisors would persuade those who had no urgent matters to deal with to return home, register the information of the villagers who had urgent business and allow them to leave the village. For those who wanted to enter the village, the supervisors would persuade them to leave. Fourth, all the village cadres, doctors, volunteers, and other staff on the frontline of COVID-19 prevention and control in the villages were equipped with masks and disinfectant. This could not only protect themselves and the people whom they came near but also played a leading role in protecting the villagers. Fifth, the village cadres arranged personnel to patrol the village and conducted key supervision of the areas where villagers could easily gather to prevent them from doing so. Those who repeatedly gathered in defiance of the exhortations were exposed through loudspeakers and social media. Those who obstructed and undermined the collective action for COVID-19 prevention and control were systematically punished. Generally speaking, village cadres had taken various COVID-19 prevention and control measures, which were the embodiment of public leadership. This ultimately had a positive impact on the villagers’ collective action for COVID-19 prevention and control.

Another aspect that must not be forgotten is the positive roles that the distance from villagers’ home village to the county and the publicity measures, which are control variables in our models, played in collective action for COVID-19 prevention and control. On the one hand, the villages close to the county have a relatively large human flow and dense population. In this case, villagers are at high risk of COVID-19 infection. Fortunately, since these villagers are near to the county’s political and economic center, they have higher awareness of epidemic prevention and better access to medical resources, such as adequate supplies of masks and timely medical assistance. These factors contributed to their active epidemic prevention and control behaviors. On the other hand, villages used a variety of ways to publicize COVID-19 prevention and control measures. For example, village cadres and doctors publicized epidemic prevention and control instructions and popular science knowledge through loudspeakers and social media. Eye-catching red banners were hung in villages to remind villagers to take the epidemic seriously. All of these publicity measures can help villagers to understand better the severity of the COVID-19 outbreak, thereby minimizing infections and avoiding respiratory transmission.

In view of the roles of moral obligation and public leadership in promoting villagers’ participation in collective action for epidemic prevention and control, the following policy implications can be proposed. On the one hand, moral obligation is an inherent restraint mechanism for villagers [52] and a powerful supplement to the institutions and rules. However, institutions and rules can be internalized into villagers’ morals, which is a significant path to improving their moral obligation. Specifically, the village cadres could put effort into formulating a village public health governance scheme with the effective participation of the villagers. Then, the villagers can master this scheme in various ways, which could help them to internalize the epidemic prevention and control rules into their moral obligation [53]. On the other hand, village cadres’ public leadership, as an external constraint for villagers, can effectively motivate villagers to participate in collective action for epidemic prevention and control. However, due to the talent shortage caused by the rural population outflow, the supply of rural public leadership is seriously insufficient. Therefore, the government needs to improve the supply of public leadership in the village in various ways. Specifically, the following measures can be taken: firstly, strengthen the training of the existing village cadres, improve the awareness and ability of village cadres to govern villages and thereby effectively promote their public leadership; secondly, encourage village elites to devote their leadership actively to the governance of village public affairs and drive other villagers to participate; thirdly, encourage elites to run for village cadres and then transform their personal leadership into public leadership; last, but not least, implement policies to attract village elites who work outside the village to return to their village, thereby increasing the village’s human capital, preparing talent reserves for the village’s public leadership, and increasing the village’s public leadership supply.

The main innovation of this article is to introduce villagers’ moral obligation and village cadres’ public leadership into the research on the collective action for epidemic prevention and control. It explores the influence mechanism of moral obligation and public leadership on villagers’ participation in collective action for epidemic prevention and control. Besides, this article creatively explores the interactive relationship between moral obligations and public leadership and its impact on collective action which previous research overlooks. These research conclusions can help villages to promote better collective action in public health governance. Unfortunately, our study area is just a rural area in Henan province, so it cannot fully represent the general situation in China.

## 7. Conclusions

In rural China, epidemic prevention and control mainly depend on villagers’ collective action. Thus, it is essential to explore the key factors influencing collective action. During the corona virus disease 2019 (COVID-19) epidemic, villagers’ epidemic prevention and control behavior was mainly influenced by internal morality and the external epidemic prevention and control scheme. However, whether the scheme could be implemented effectively depended mainly on the leadership of village cadres. Therefore, this paper focuses on the impact of villagers’ moral obligation and village cadres’ public leadership on collective action for COVID-19 prevention and control in rural China. Based on survey data from 533 villagers of Henan province, this paper employed the principal component analysis (PCA) to simplify the multidimensional information of moral obligation and public leadership and ordered probit regression to analyze their influence on collective action for COVID-19 prevention and control. The control variables selected according to the institutional analysis and development (IAD) framework were also considered for analysis. The results showed that moral obligation, public leadership and their interaction had significantly positive effects on collective action for COVID-19 prevention and control. Additionally, the distance from villagers’ home villages to the county and publicity measures, which are control variables, played a positive role.

The main innovation of this paper is to introduce moral obligation and public leadership into collective action for epidemic prevention and control. Moreover, this paper innovatively explores the interaction effects of moral obligation and public leadership on collective action, which previous studies neglect. In addition, the findings of this paper indicate that it is necessary to develop village epidemic response plans that could enhance moral obligation and public leadership in villages. For example, the government could improve village cadres’ public leadership level by training existing village cadres and encouraging village elites to run for village cadres. Furthermore, with the effective participation of villagers, village cadres should formulate and constantly improve their village’s epidemic response plan. Village cadres could organize villagers to master this response plan in various ways to help them internalize the code of conduct in the plan into standards of moral behavior during epidemics. Once an epidemic occurs, village cadres could initiate the response plan in time and exert their leadership to implement the measures effectively. Besides, villagers could take positive action to prevent the epidemic under moral constraints to realize collective action for epidemic prevention and control. However, since the Chinese rural governance system is unique, research on collective action for epidemic prevention and control and the effects of moral obligation and public leadership on it in other regions of the world should be further verified.

## Figures and Tables

**Table 1:** Definitions and summary statistics of the variables.

Variable	Definition	Mean	SD	Min.	Max.
*Collective Action*					
Going out	Frequency of going out: 1—at least once per day; 2—once every 2–3 days; 3—once every 4–5 days; 4—once every 6–7 days; 5—once every 8 days or more	3.627	0.935	1	5
Wearing masks	Wearing masks when going out: 1 (never); ~5 (every time)	2.852	1.079	1	5
*Moral Obligation*					
Sense of obligation	I feel morally obliged to participate in COVID-19 prevention and control: 1 (totally disagree); 5 (totally agree)	3.947	0.970	1	5
Personal satisfaction	I feel satisfied participating in COVID-19 prevention and control: 1 (totally disagree); 5 (totally agree)	4.077	0.876	1	5
Autonomy	Whatever others may think, I will participate in COVID-19 prevention and control: 1 (totally disagree); 5 (totally agree)	3.927	0.924	1	5
Objectivity	I will participate in COVID-19 prevention and control because this is clearly a positive measure: 1 (totally disagree); 5 (totally agree)	4.242	0.783	1	5
*Public Leadership*					
Influence force	Village cadres have adequate influence force: 1 (totally disagree); 5 (totally agree)	3.233	0.851	1	5
Decision-making ability	Village cadres can coordinate the interests of all the stakeholders in the decision-making process of COVID-19 prevention and control: 1 (totally disagree); 5 (totally agree)	3.362	0.815	1	5
Executive ability	Village cadres can efficiently implement the measures of COVID-19 prevention and control: 1 (totally disagree); 5 (totally agree)	3.540	0.721	1	5
Creativity	Village cadres can make a creative and effective scheme for COVID-19 prevention and control: 1 (totally disagree); 5 (totally agree)	3.700	0.795	1	5
*Physical Condition*					
Distance to the county	Distance from the village to the county: 1—more than 40 km; 2—more than 30 km and less than or equal to 40 km; 3—more than 20 km and less than or equal to 30 km; 4—more than 10 km and less than or equal to 20 km; 5—less than or equal to 10 km	2.593	1.021	1	5
*Household Characteristics*					
Age	Villager’s age in years	42.993	8.226	19	62
Education	Education level: 1—primary school or below, 2—middle school, 3—high school, 4—college/university, 5—graduate school or above	2.107	0.700	1	5
Child or elder	Whether a child (under 15 years old) or elderly person (over 60 years old) is living in the home: 1—yes; 0—no	0.323	0.468	0	1
*Rules-in-Use*					
Publicity	Publicity measures for COVID-19 prevention and control: 1 (none); 5 (sufficient)	3.782	0.866	1	5
Supervision	Supervision mechanism for COVID-19 prevention and control: 1 (completely useless); 5 (efficient)	3.538	0.900	1	5
Punishment	Punishment mechanism for COVID-19 prevention and control: 1 (completely useless); 5 (efficient)	3.702	1.102	1	5

**Table 2:** Outcome of the reliability and validity analysis.

Composites/Indicators	KMO	Bartlett’s Test	Factor Loading	CPV (%)	Cronbach’s Alpha	CR	AVE
*Moral Obligation*	0.738	795.387 (0.000)		64.962	0.817	0.881	0.650
Sense of obligation			0.838				
Personal satisfaction			0.788				
Autonomy			0.771				
Objectivity			0.825				
*Public Leadership*	0.751	736.929 (0.000)		63.524	0.803	0.874	0.635
Influence force			0.809				
Decision-making ability			0.794				
Executive ability			0.871				
Creativity			0.705				

**Table 3:** Multicollinearity diagnosis.

	Variable	Collinearity Statistics			
		1/VIF	VIF	1/VIF	VIF
Distance to the county	*Moral Obligation*				
	General moral obligation	0.473	2.116		
	Sense of obligation			0.439	2.278
	Personal satisfaction			0.528	1.895
	Autonomy			0.485	2.061
	Objectivity			0.436	2.294
	*Public Leadership*				
	General public leadership	0.616	1.622		
	Influence force			0.513	1.950
	Decision-making ability			0.361	2.773
	Executive ability			0.427	2.342
	Creativity			0.667	1.499
	*Household Characteristics*				
	Age	0.958	1.044	0.943	1.060
	Education	0.973	1.028	0.968	1.033
	Child or elderly person	0.982	1.019	0.974	1.026
	*Rules-in-Use*				
	Publicity	0.700	1.430	0.682	1.467
	Supervision	0.460	2.176	0.398	2.510
	Punishment	0.439	2.280	0.414	2.418

**Table 4:** The effects of moral obligation and public leadership on villagers’ going out frequency.

Variable	Model 1	Model 2	Model 3	Model 4
*Moral Obligation*				
General moral obligation	0.420 *** (0.054)	0.291 *** (0.071)		0.329 *** (0.072)
Sense of obligation			0.065 (0.076)	
Personal satisfaction			0.017 (0.077)	
Autonomy			0.129 * (0.075)	
Objectivity			0.210 ** (0.094)	
*Public Leadership*				
General public leadership	0.389 *** (0.054)	0.214 *** (0.067)		0.251 *** (0.069)
Influence force			0.041 (0.084)	
Decision-making ability			0.101 (0.099)	
Executive ability			0.191 * (0.103)	
Creativity			0.016 (0.075)	
*Interaction Item*				
General moral obligation * General public leadership				0.141 *** (0.046)
*Physical Condition*				
Distance to the county		0.211 *** (0.056)	0.232 *** (0.059)	0.200 *** (0.056)
*Household Characteristics*				
Age		−0.003 (0.006)	−0.004 (0.006)	−0.003 (0.006)
Education		−0.041 (0.071)	−0.048 (0.071)	−0.051 (0.071)
Child or elderly person		0.006 (0.106)	0.007 (0.106)	0.039 (0.107)
*Rules-in-Use*				
Publicity		0.197 *** (0.068)	0.193 *** (0.068)	0.189 *** (0.068)
Supervision		0.087 (0.080)	0.075 (0.086)	0.078 (0.081)
Punishment		0.061 (0.067)	0.050 (0.069)	0.071 (0.067)
Number of observations	533	533	533	533
LR χ^2^	168.02	196.18	199.05	205.60
Prob > χ^2^	0.0000	0.0000	0.0000	0.0000
Pseudo R^2^	0.1282	0.1496	0.1518	0.1568
Log likelihood	−571.5180	−557.4400	−556.0010	−552.7296

Note: Standard errors are reported in parentheses. *** *p* < 0.01; ** *p* < 0.05; * *p* < 0.1.

**Table 5:** The effects of moral obligation and public leadership on villagers’ frequency of wearing a mask when going out.

Variable	Model 5	Model 6	Model 7	Model 8
*Moral Obligation*				
General moral obligation	0.273 *** (0.051)	0.228 *** (0.069)		0.251 *** (0.069)
Sense of obligation			0.053 (0.074)	
Personal satisfaction			0.050 (0.074)	
Autonomy			0.159 ** (0.073)	
Objectivity			0.085 (0.092)	
*Public Leadership*				
General public leadership	0.517 *** (0.054)	0.355 *** (0.065)		0.381 *** (0.066)
Influence force			0.253 *** (0.082)	
Decision-making ability			0.174 * (0.096)	
Executive ability			0.048 (0.099)	
Creativity			0.073 (0.073)	
*Interaction Item*				
General moral obligation * General public leadership				0.108 ** (0.045)
*Physical Condition*				
Distance to the county		0.307 *** (0.053)	0.280 *** (0.056)	0.297 *** (0.053)
*Household Characteristics*				
Age		0.005 (0.006)	0.003 (0.006)	0.005 (0.006)
Education		0.006 (0.068)	0.008 (0.068)	0.001 (0.068)
Child or elderly person		−0.008 (0.100)	−0.020 (0.100)	0.013 (0.101)
*Rules-in-Use*				
Publicity		0.080 (0.065)	0.087 (0.066)	0.072 (0.065)
Supervision		0.093 (0.077)	0.082 (0.082)	0.088 (0.077)
Punishment		−0.026 (0.064)	−0.027 (0.066)	−0.023 (0.064)
Number of observations	533	533	533	533
LR χ^2^	174.92	211.68	216.60	217.49
Prob > χ^2^	0.0000	0.0000	0.0000	0.0000
Pseudo R^2^	0.1139	0.1379	0.1411	0.1417
Log likelihood	−680.1534	−661.7778	−659.3132	−658.8722

Note: Standard errors are reported in parentheses. *** *p* < 0.01; ** *p* < 0.05; * *p* < 0.1.
